# Scooping Technique to Acquire Cancellous Bone for Grafting in the Masquelet Procedure: A Retrospective Study

**DOI:** 10.1007/s43465-023-00909-3

**Published:** 2023-05-24

**Authors:** Hui Wang, Zhihong Zhang, Wanming Wang, Xiaotang Sun

**Affiliations:** 1 Department of Orthopedics Surgery, The 900th Hospital of Joint Logistics Support Force, PLA 156 West Second Ring North Road, Gulou District, Fuzhou, 350025 Fujian China; 2 Fuzong Clinical Medical College of Fujian Medical University, 88 Jiaotong Road, Taijiang District, Fuzhou, 350025 Fujian China

**Keywords:** Scooping, Iliac crest, Grafting, Cancellous bone, Autogenous, Masquelet, Retrospective study, Bone defect, Minimally invasive, Bone regeneration

## Abstract

**Background:**

The Masquelet procedure is effective in overcoming large bone defects; however, the limited number of cancellous bone and donor site complications remains a challenge. We developed a scooping technique to harvest sufficient cancellous bone from iliac crests for grafting during the Masquelet procedure. We hypothesized that this method would be efficient and safe.

**Methods:**

This retrospective study included 13 patients who underwent the Masquelet procedure with cancellous bone grafting using the scooping technique. The following parameters were observed: (1) duration and total volume of cancellous bone extraction; (2) amount of bleeding and drainage fluid, and Visual Analog Scale (VAS) score of pain at the donor site during different periods; and (3) complications and bone regeneration at the ilium at the final follow-up.

**Results:**

The median follow-up duration was 17 months. There were 3 unilateral and 10 bilateral extraction sites. The mean total amount extracted, extraction duration, bleeding, and drainage were 39 mL, 23 min, 49 mL, and 44 mL, respectively. Only three patients felt pain (VAS score: 1 point) at the final follow-up. Postoperatively, one case each of hematoma and lateral femoral cutaneous nerve injury supervened, and no infections or other complications occurred. The last computed tomography examination showed varying degrees of bone regeneration in the ilium.

**Conclusion:**

The scooping technique for the iliac crest produced a substantial amount of autogenous cancellous bone using a small incision. It retained the appearance and morphology of the ilium with few complications. We believe it is a successful and safe option for treating bone defects.

## Introduction

Masquelet et al. [1] reported the reconstruction of large diaphyseal defects in two stages. The first stage involves resectioning the infected bone segment and interposing polymethylmethacrylate (PMMA). The second stage involves removing PMMA and implanting a large fresh autologous cancellous bone graft within the membrane for bone defect reconstruction. This technique is effective and can shorten the therapeutic course. Recently, it has gained acceptance among orthopedic surgeons for acute traumatic bone defects [2, 3]. However, the defect size is often large in both post-traumatic and post-excision infected bone defects. Reportedly, some bone defects are larger than 15 cm [4]. Generally, bone graft size is positively correlated with bone defects. In selecting bone grafting materials, autologous bone has an irreplaceable biological and immunological advantage over allogeneic and artificial bone and is more widely used in clinical practice. Some reports have shown that recombinant human bone morphogenetic protein-2 (RhBMP-2) can be used as an additive to promote osteogenesis. However, its clinical application is limited because of its high cost [5].

Owing to the abundance of cancellous and tri-partite cortical bone, the ilium is widely used as an autologous bone donor in orthopedics and maxillofacial orthopedics [6]. However, complications associated with bone extraction, such as donor site pain, lateral femoral cutaneous nerve injury, and local deformity, should not be ignored. Obtaining sufficient autologous cancellous bone using a safe and minimally invasive technique has also become a hot research topic. This study aimed to present a minimally invasive iliac crest scooping technique (MIICST) to obtain autologous cancellous bone using the Masquelet technique.

## Materials and Methods

### Inclusion and Exclusion Criteria

The inclusion criteria were as follows: (1) bone defects caused by infection or acute trauma treated with the Masquelet technique; (2) age < 60 years; and (3) preoperative bone density test *T* > − 2.5 SD.

The exclusion criteria were as follows: (1) diabetes or cardiovascular disease requiring long-term anticoagulant medication, (2) immune system disease requiring long-term hormone use, and (3) multiple injuries requiring long-term bed rest.

### Patients’ Information

We retrospectively analyzed the outcomes of 13 patients (13 sides) who underwent the Masquelet technique for bone defects at our hospital between October 2018 and September 2020. Of these, nine were males and four were females, aged 22–52 (38.42 ± 9.69) years; there were five femoral bone defects, seven tibial bone defects, and one humerus bone defect. Three bone defects were attributed to trauma, and 10 were caused by infection.

### Surgical Procedure

According to Masquelet’s original procedure, all patients were treated with PMMA interposition in phase I and autologous cancellous bone grafting in phase II, when the local soft tissue conditions were stable. MIICST was used for the extraction. Other combined procedures, include fracture reduction and lesion debridement. This study was approved by the institutional review board of the authors’ affiliated institutions.

### MIICST

**Fig. 1:**
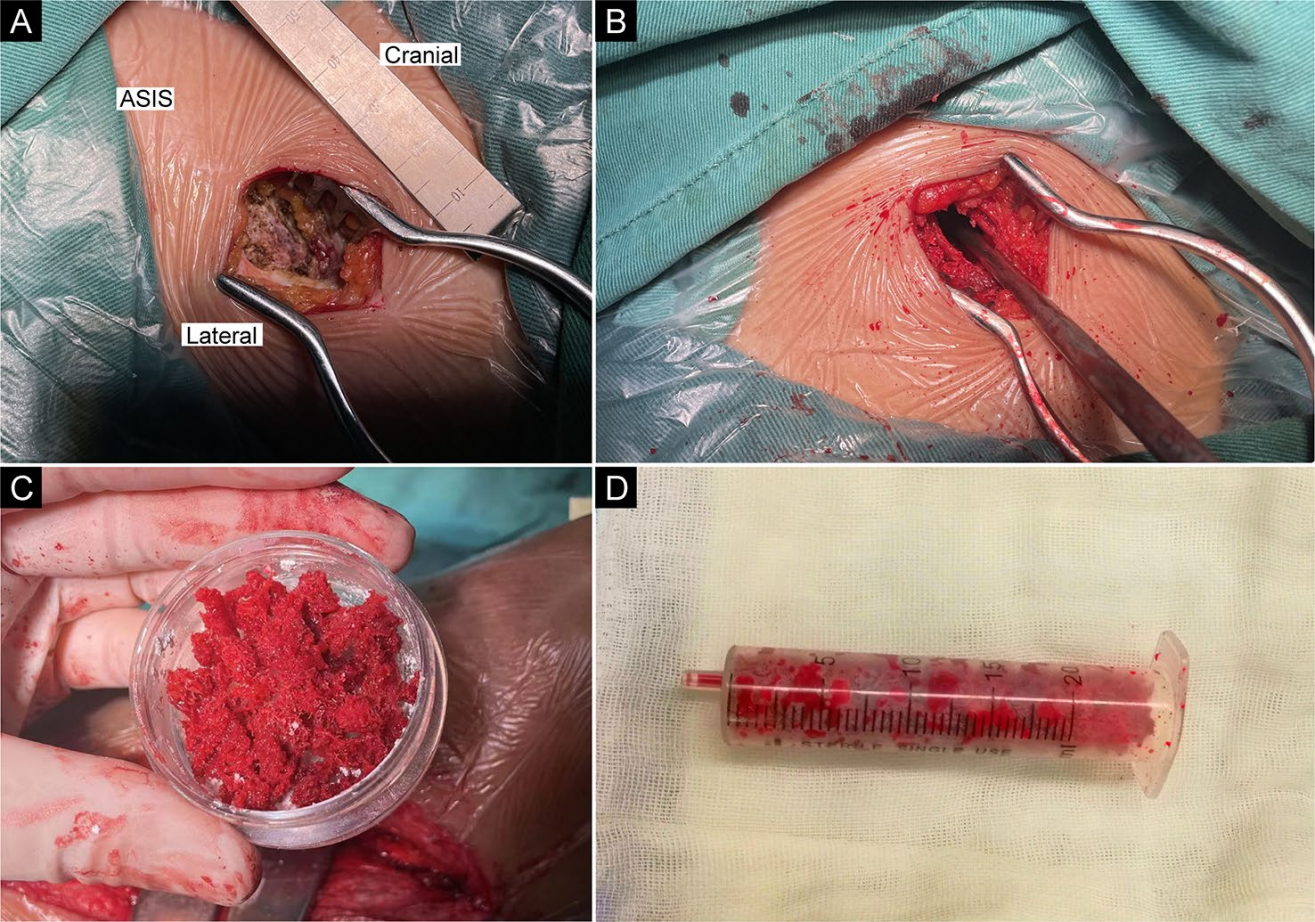
**a** Skin incision. The approximate 2.5 cm skin incision was made 2 cm behind the ASIS. **b** The nucleus pulposus forceps and scraper were used to harvest cancellous bone between the medial and lateral plates of the iliac bone, similar to a tunnel. **c, d** The cancellous bone obtained was placed in a syringe to assess the bone volume. ASIS, anterior superior iliac spine

**Fig. 2:**
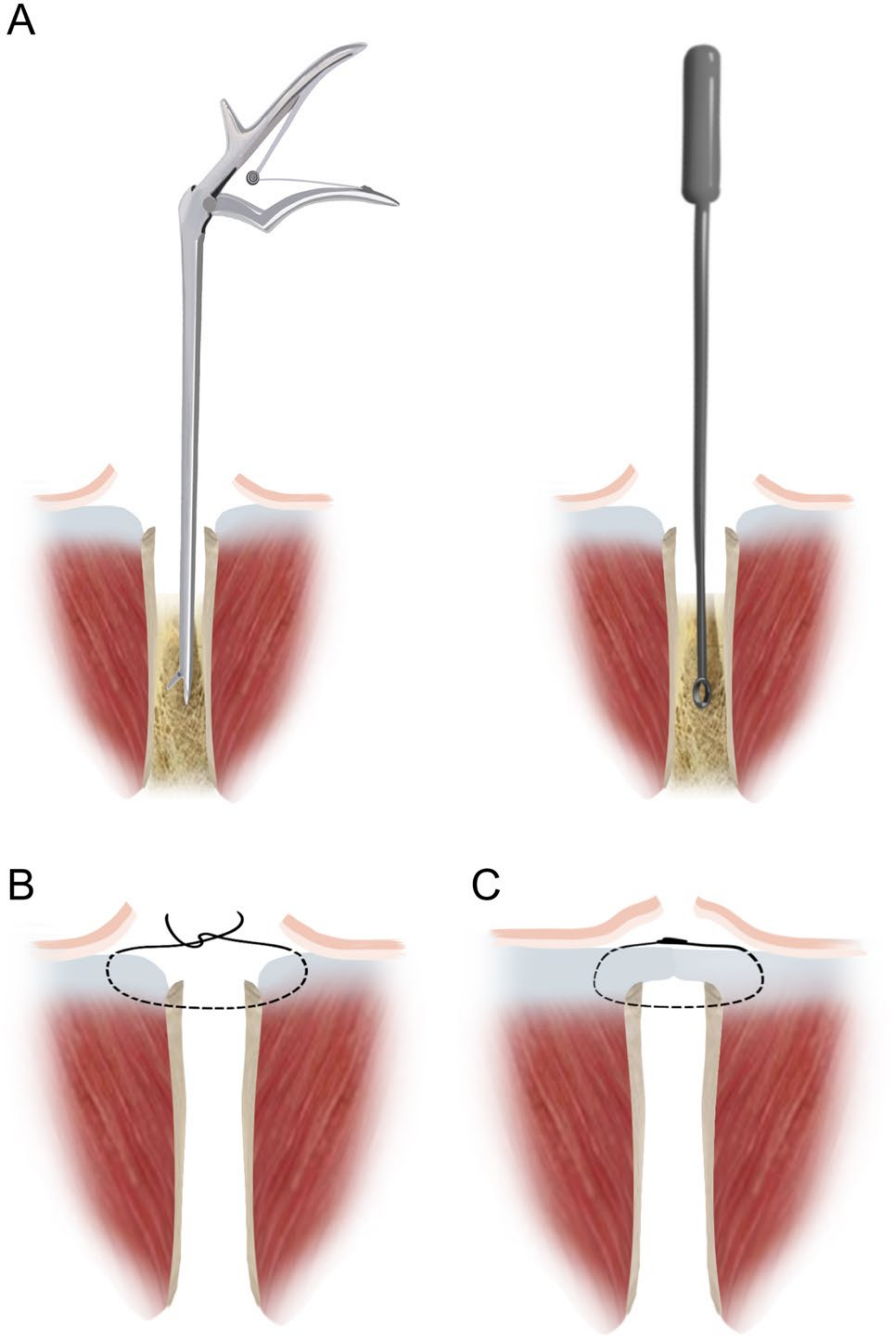
**a** A schematic diagram showing how to harvest the cancellous bone with the nucleus pulposus forceps and scraper; **b, c** The method and level of the suture

The patients were placed supine under combined spinal–epidural anesthesia, and upper limb surgery was performed under brachial plexus anesthesia. The affected limb and bilateral iliac areas were routinely disinfected and draped. The anterior superior iliac spine (ASIS) was palpated on the body surface. An approximate 25 mm incision was made along the iliac crest, 20 mm posterior to the ASIS (intraoperative photographs Fig. 1a). We made a spot on the surface of the iliac crest using an awl. We extended it to approximately 10–15 mm using different models of nucleus pulposus forceps and a scraper (1–3 mm in diameter) (Qing Niu, Suzhou, Jiang Su Province, China). Moreover, a diameter scraper was used to remove some of the cortical bone and a small amount of cancellous bone from the superficial to deep level (schematic diagram Fig. 2a). The cancellous bone was clamped using different models of the nucleus pulposus forceps and scraper between the medial and lateral plates of the iliac bone, similar to a tunnel (intraoperative photographs Fig. 1b). Care was taken to prevent bleeding within the iliac muscle due to penetration of the medial and lateral iliac plates using nucleus pulposus forceps. Hemostasis sponges mixed with 1 g of tranexamic acid were filled into the tunnel cavity to reduce bleeding, and a drainage tube was placed if necessary. The drainage was released after clamping for 6 h. Bone wax was avoided because it could affect osteogenesis in the future. The incision was sutured (schematic diagrams Fig. 2b, c), and local pressure was applied appropriately at the end. The drainage tube was removed if the fluid volume was < 30 mL/day. The sutures in the iliac region were removed after 1 week.

After removing the PMMA, we assessed the level of the bone defect (intraoperative photographs Fig. 1c, d). If the cancellous bone from one site was insufficient, the contralateral site was added. If the total amount of bone obtained was insufficient, a homogeneous allograft or artificial bone was added, but not more than 30% of the total bone graft.

### Evaluation Items

The following seven items were evaluated: (1) duration of cancellous bone extraction (from skin incision to the end of suturing); (2) total volume of cancellous bone extracted [in a 50 mL syringe; care was taken to avoid deliberate compression of the cancellous bone (intraoperative photographs 2)]; (3) amount of bleeding; (4) the total amount of drainage fluid; (5) Visual Analog Scale (VAS) score of pain in the iliac region after 1 day, 1 month, and at the final follow-up examination; (6) corresponding complications (such as infection, hematoma, lateral femoral cutaneous nerve injury); and (7) computed tomography (CT) at the final follow-up to evaluate bone regeneration in the donor site.

## Results

Patient characteristics are summarized in Table 1. Iliac morphology was intact in all patients. Unilateral and bilateral bone extractions were performed in 3 and 10 patients, respectively. The total volume of bone extracted was 39.00 ± 12.64 mL, while the extraction duration was 23.00 ± 11.16 min; the amount of bleeding during surgery was 49.00 ± 27.60 mL, and the total postoperative drainage was 44.00 ± 29.84 mL. All patients were followed up, and the median follow-up duration was 17 months.

**Table 1:** Characteristics of the 13 patients with bone defects

Patient	Age		BMI	Sex	Bone	Reason	AO-Classification	Fracture Fixation	Bone Extraction Volume, mL	Extraction Time, min	Extraction Bleeding, mL	Drainage, mL	VAS			Integrity of Cortical Ilium Body	Defect Filling	Complication	Autologous Bone Harvest Site	Bone Graft	Bone reconstruction in Ilium	Follow-up Month
													1D	1 M	LAST							
1	52		28.7	M	R Femur	Trauma	33-C3	Plate (Lat + Med)	57	32	95	80	3	1	0	Yes	HS + TA	None	Bilateral	Autol + Allog (8 mL) + PMMA column	(+)	37
2	18		19.1	M	R Tibia	Infection	/	Nail	33	25	65	48	2	0	0	Yes	HS + TA	None	Bilateral	Autol	(+)	6
3	45		28	F	R Tibia	Infection	/	Nail	50	38	74	55	2	1	1	No	HS + TA	None	Bilateral	Autol	(+)	31
4	20		16.9	M	L Femur	Infection	/	Plate (Lat + Med)	40	30	45	18	2	0	0	Yes	HS + TA	None	Bilateral	Autol + Allog (8 mL)	(+)	17
5	32		23.1	M	R Femur	Infection	/	Nail	35	12	28	45	1	0	0	Yes	HS + TA	None	Bilateral	Autol	(+)	23
6	48		23.9	M	L Femur	Infection	/	Plate (Lat)	58	40	90	105	4	1	1	No	HS + TA	Hematoma	Bilateral	Autol + Allog (8 mL)	(+)	14
7	45		21.3	M	L Tibia	Infection	/	/	40	16	28	42	1	0	0	Yes	HS + TA	None	Bilateral	Autol	(+)	7
8	53		25	M	L Tibia	Infection	/	Plate (Lat)	26	7	15	12	2	1	1	Yes	HS	None	Unilateral	Autol	(+)	25
9	41		24.2	M	R Tibia	Infection	/	Plate (Lat)	46	26	69	65	3	1	0	Yes	HS + TA	Irritation of LFN	Bilateral	Autol	(+)	14
10	43		24.7	F	R Humerus	Infection	/	Plate (Lat + Med)	38	31	46	58	2	1	0	Yes	HS + TA	None	Bilateral	Autol	(+)	33
11	35		18.6	M	L Tibia	Trauma	42-C2	Plate (Med)	24	16	20	10	2	0	0	Yes	HS	None	Unilateral	Autol	(+)	22
12	68		22.4	F	L Femur	Trauma	33-C2	Plate (Lat + Med)	45	20	47	34	3	0	0	Yes	HS + TA	None	Bilateral	Autol	(+)	14
13	69		30.9	F	L Tibia	Infection	/	Plate (Lat)	15	6	15	0	1	0	0	Yes	HS	None	Unilateral	Autol	(+)	11

R, right; L, left; Lat, lateral; Med, medium; LFN, lateral femoral nerve; Autol, autologous; Allog, allograft; HS, Hemostasis sponges; TA, tranexamic acid

VAS scores were available for all patients at the postoperative follow-up. The mean VAS score was 2.15 [standard deviation (SD): 0.90] points on 1 day, 0.46 (SD: 0.52) points after 1 month, and 0.31 (SD: 0.48) points at the final follow-up. At the last follow-up examination, three patients had a VAS score of only 1 point for donor site pain that did not interfere with sleep, hip flexion, sitting, and standing. Ecchymosis and hematoma appeared locally in only one patient; the patient had a VAS score of 4 points on postoperative day 1 following bilateral iliac extraction. The bleeding volume was 90 mL, and the drainage volume was 105 mL. This patient was treated with nonsteroidal anti-inflammatory drugs (NSAIDs) and had a VAS score of 2 points at 72 h postoperatively, which was tolerable.

MIICST was used for iliac crest bone extraction in all patients, although the bone volume was insufficient in some. Owing to the large bone defects in three cases, cancellous bone was extracted from the bilateral iliac bone (57, 40, and 58 mL), which could not fulfill the requirement for grafting. Thus, we added 8 mL of allograft bone in these three cases. Complying with the previous studies [7, 8], the added allograft did not exceed 30% of the total bone graft amount. A large bone defect remained in one case; therefore, a long column of PMMA was prepared to fill the middle of the bone and improve the stability of the graft.

**Fig. 3:**
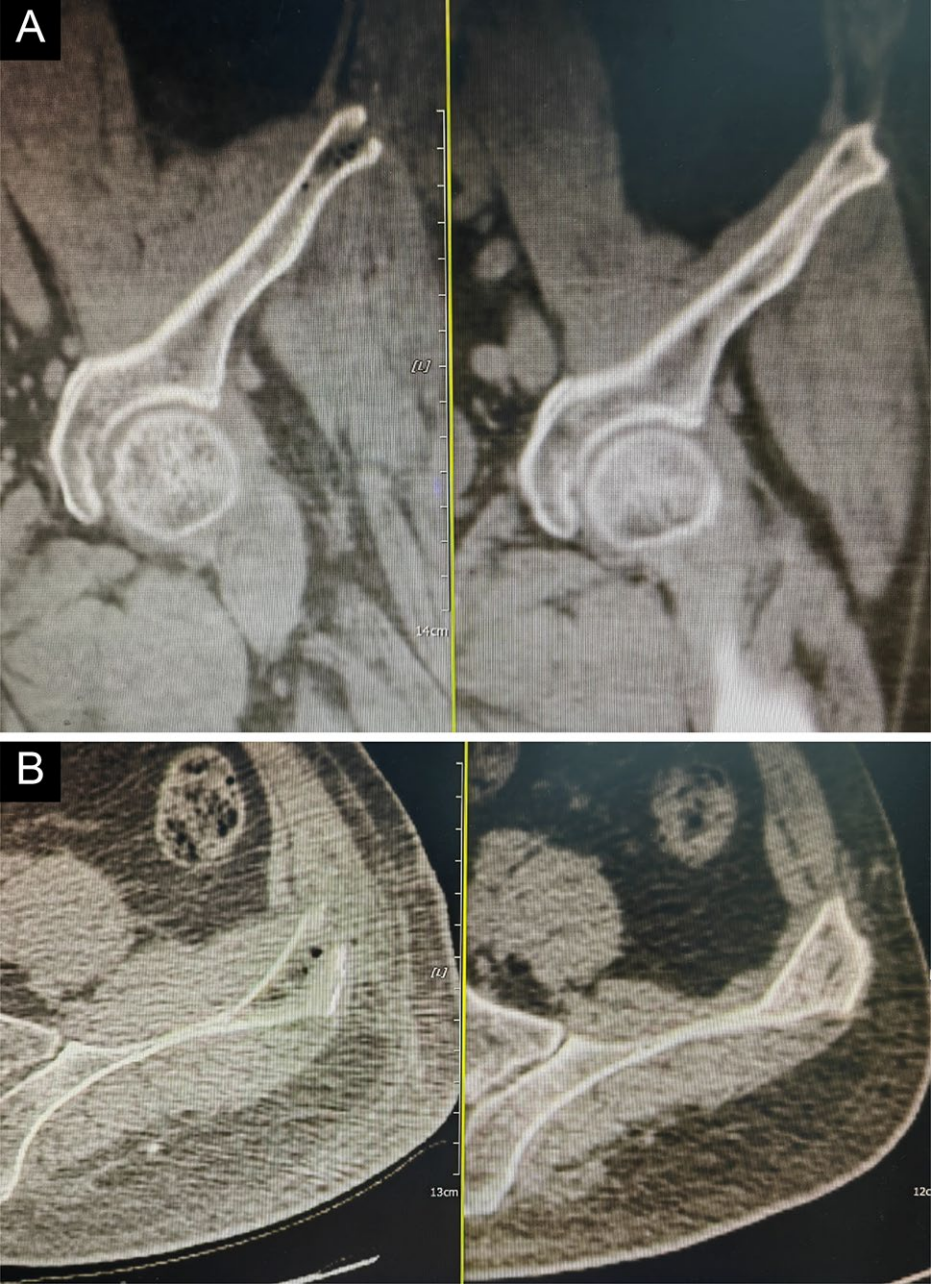
**a** The immediate morphology of the iliac crest after MIICST. **b** Bone regeneration at the last follow-up. MIICST, minimally invasive iliac crest scooping technique

All wounds healed smoothly, with no infections, iatrogenic fractures, or heterotopic ossification. One patient developed a local hematoma, whereas another experienced irritation of the lateral femoral nerve, which disappeared after 2 weeks. CT examination at the last follow-up revealed different degrees of bone regeneration in the ilium (Fig. 3a, b).

## Case Example

**Fig. 4:**
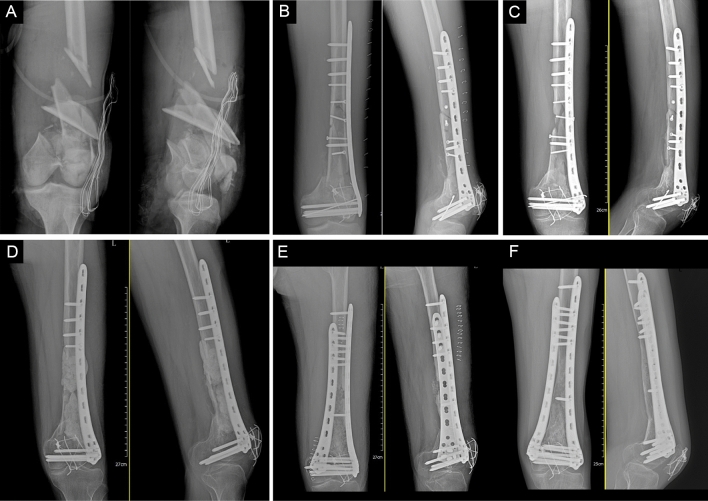
**a** Anteroposterior and lateral radiographs of the left femoral of a 20-year-old male injured in a car accident. The patient experienced an open comminuted fracture of the distal femur. **b** Anteroposterior and lateral radiographs of the left femur after lateral plate. **c** Anteroposterior and lateral radiographs of the left femur after half a year. Unfortunately, a localized infection developed and formed a sinus tract, and the fracture did not heal. **d** Anteroposterior and lateral radiographs of the left femur after bone debridement and placement of a polymethylmethacrylate spacer in the femoral bone defect. **e** Anteroposterior and lateral radiographs of the left femur after bone grafting. **f** Anteroposterior and lateral radiographs of the left femur taken 17 months postoperatively show a mature bone callus

A 20-year-old male suffered an open comminuted fracture of the distal left femur in a car accident and underwent debridement, open reduction, and internal fixation of fractures. Unfortunately, his wound became infected, and a sinus formed. Six months later, we applied the Masquelet technique to treat the bone infection. After 2 months, we removed the PMMA, used MIICST to obtain approximately 40 mL of autogenous cancellous bone from bilateral sites, and mixed 8 mL of allograft bone to fill the bone defect area. Furthermore, we used lateral and medium plates to provide bone stability. After 17 months of follow-up, the infection was cured, and the bone healed well (Fig. 4a–f).

## Discussion

### Characteristics of Autologous Bone Graft

After more than 100 years of clinical application, autologous bone grafts remain the gold standard for treating bone defects because they are safe and effective, even with the rapid development of material science [9, 10]. Approximately 2 million graft-related surgeries are performed worldwide each year. Autologous bone has three properties: osteogenesis, osteoinduction, and osteoconduction [11]. Autologous bone can be divided into cortical and cancellous bone, and vascularized and nonvascular bone. It can be selected according to the transplantation site needs. However, cancellous bone, rich in osteogenic stem cells and growth factors, has better osteogenic properties than cortical bone. Although cancellous bone grafts provide poor initial structural support, initial stability can be achieved with metallic materials, such as internal fixation.

The most commonly used areas for autologous bone extraction are the anterior ilium, posterior ilium, femoral bone marrow cavity, anterior tibial region, and olecranon. The disadvantages of autologous bone harvesting are also apparent, such as additional surgical trauma, bleeding, operation time, and complications. These complications include pain, hematoma, hernia, and loss of sensation, and are directly related to the bone harvesting site [12]. The iliac bone, especially the anterior iliac crest (AIC), has always been the first clinical choice for obtaining autogenous bone owing to its anatomically superficial location, large volume, and ease of retrieval [13]. There may be fewer complications associated with the use of the posterior iliac crest (PIC) than with AIC [14]. However, PIC requires a prone position, which is inconsistent with most surgical positions. Hence, its application is scarcer than that of AIC. Le Baron et al. [15] considered the Reamer Irrigator Aspirator system expensive; they could obtain a certain amount of cancellous bone from the femoral bone marrow cavity and had fewer complications than the procedure to obtain cancellous bone from the iliac crest. Burk et al. [16], through a cadaver study, showed that osteotomy 2.5 cm beyond the tibial plateau and 1.5 cm behind the tibia could obtain approximately 18 mL of uncompressed cancellous bone. However, this operation has the disadvantages of postoperative platform collapse and early weight-bearing failure due to pain and local bone mechanical changes.

### Analysis of the Difference Among the Iliac Crest Bone Extraction Methods

Various iliac bone extraction methods have different merits and demerits. The traditional open “U” bone extraction, although effective for obtaining a large amount of cancellous and cortical bone, inevitably result in complications, such as permanent bone loss, herniation in the donor area, residual cosmetic deformity, nerve damage, and local pain. Materials used to repair iliac bone defects include screws and PMMA, a flexible titanium craniofacial reconstruction plate, a polypropylene/poliglecaprone parietal reinforcement prosthesis, hydroxyapatite–calcium triphosphate biphasic compound, and bioactive ceramic spacers [17, 18]. However, we believe this procedure increased surgical costs and was limited by late local loosening and permanent bone defects in the iliac region.

Gil-Albarova et al. [19] reported a tri-cortical bone extraction from the iliac crest followed by excision of a portion of the iliac bone into a fenestrated shape and replantation in the extraction area could be used to replicate the appearance of the ilium. However, the procedure is cumbersome, and the amount of bone obtained is limited. Recently, the trephine graft harvest technique was designed specifically for iliac bone extraction [20]; however, the risk of local hematoma exists in this blind method owing to the penetration of the iliac cortex and damage to the muscle tissue. Moreover, its clinical application is limited. Some surgeons do not pay much attention to the extraction method, which leads to postoperative pain in the extraction area. Kim et al. [21] observed that approximately half of the patients experienced acute postoperative pain, while the incidence of chronic pain was as high as 13%. Some modifications in iliac bone harvesting can minimize donor site pain [22, 23].

Evolving surgical methods are designed to be minimally invasive. Lopez et al. [22] used a high-speed burr to penetrate the cortex of the PIC and used the core reamer and straight and curved curettes to harvest the cancellous bone. We believe that a Kerrison rongeur could be an ideal instrument to enlarge the hole in the iliac crest made by the awl, and this would be minimally invasive compared to a high-speed burr. Some companies offer trephine tools for iliac crest harvesting; however, their drilled bone channels are larger and are more likely to cause damage to the inner and outer plates of the iliac crest [24]. Shaw et al. [25] presented the same surgical technique for harvesting the cancellous bone graft using curets only. We used different models of nucleus pulposus forceps and scrapers to operate, resulting in better efficiency.

Salawu et al. [26] reported that the average compressed volume of graft harvested from the iliac crest was 5.5 cm^3^. Kessler et al. [27] harvested 127 bone grafts from the iliac crest and found that the mean volume of bone harvested was 15 cm^3^. In our research, the average noncompressed bone volumes harvested unilaterally and bilaterally were 21.6 and 44.2 mL, respectively. The Masquelet technique does not require compressed cancellous bone for grafting. Therefore, syringes were used to measure the approximate volume without deliberately compressing the cancellous bone. This is why our volumes are much larger than those reported in the literature.

### Features of MIICST

MIICST uses different scrapers and nucleus pulposus forceps inside the iliac bone through a longitudinal window on the iliac crest surface. This procedure has the following advantages: (1) The incision is small, which can retain the surrounding relatively intact bone–muscle attachment and the overall cortical bone of the iliac crest. The anatomical morphology was not damaged, and the appearance was normal. Avulsion fracture of the ASIS due to muscle pulling after surgery was avoided. (2) The broad fascia latae and gluteus medius and minimus, which are attached to the internal and external iliac plates, respectively, are not stripped (hip mobility disorder and abdominal hernia are avoided postoperatively). (3) The vacant iliac bone marrow cavity is filled with an absorbable gelatin sponge and tranexamic acid to stop bleeding by swelling the sponge [16]. The drainage tube was clamped for 6 h and removed after 24 h, making it less likely to form a hematoma. (4) The bone marrow of the iliac bone is rich in osteogenic precursor cells and morphogenetic proteins, which have strong autologous osteogenic capacity. (5) To avoid injury to other structures, the surgeon must operate gently and carefully to perceive the sensation of the scraper in the cancellous bone. This process is simple, economic, and reliable, with a short learning curve and without additional materials for reconstruction. The patients were satisfied with the small scars, lower donor site pain, and lesser abnormal feeling of stepping at the follow-up examination.

The lateral femoral cutaneous nerve originates from the anterior branch of the lumbar plexus, runs 10–15 mm below the ASIS through the inguinal ligament, and innervates the anterolateral skin. To avoid nerve damage and fracture complications in the extraction area, the cancellous bone within the iliac pterygoid was obtained by grooving approximately 20 mm behind the ASIS [28]. Although damage to the inner and outer plates was observed in some cases, stress fractures were not observed. There was one case of anterolateral femoral cutaneous nerve injury, probably due to anatomical variation of the femoral nerve, intraoperative manipulation, local hematoma, and other stimuli. However, the symptoms disappeared 2 weeks postoperatively.

We also found that some patients had relatively little bone mass in the AIC. This method can be used to harvest bone from the PIC, especially for spinal fusion surgery in a prone position. Moreover, the postoperative complications were fewer, and the clinical effect was satisfactory. We successfully treated patients with satisfying clinical effects and fewer complications. Therefore, we believe that cancellous bone can be obtained by MIICST from the AIC and PIC. Many osteogenic factors are retained in the residual cavity after bone extraction, which can also self-reconstruct a good trabecular bone in the later stage. A follow-up CT examination of the patient also confirmed this view.

According to our experience, this method of iliac crest extraction has some drawbacks. The shape of the iliac crest has a certain anatomical angle, which necessitates the surgeon’s experience. In some cases, the iliac crest has a narrow gap or sclerotic bone between the inner and outer plates, owing to which smaller nucleus pulposus forceps and scrapers are needed to avoid damage to the plates by force. This operation relies heavily on the surgeon’s tactile perception. The inner and outer plates are easily broken in patients with iliac crest osteoporosis. Nevertheless, the proposed method is economic and minimally invasive for obtaining autogenous cancellous bones.

The procedure used in this study had some limitations. It cannot meet the requirements of a procedure that requires cortical bone support in the implant area to increase stability. The amount of bone extracted was influenced by the morphology of the ilium and the amount of local cancellous bone. Preoperative CT examination may help evaluate local bone [29]. A control group was not set up, and the ability and time to reconstruct the cancellous bone after bone extraction were not observed in detail. However, we believe that this procedure has comprehensive and beneficial clinical applications owing to its small incision, simple performance, and few complications.

## Conclusion

The scooping technique is a successful and safe option for treating bone defects owing to the creation of a small incision and few associated complications. It is a good guarantee for the success of cancellous bone graft surgeries, such as the Masquelet technique.

## Data Availability

All data generated or analyzed during this study are included in this published article.
